# A Novel Human T-lymphotropic Virus Type 1c Molecular Variant in an Indigenous Individual from New Caledonia, Melanesia

**DOI:** 10.1371/journal.pntd.0005278

**Published:** 2017-01-06

**Authors:** Olivier Cassar, Françoise Charavay, Frédéric Touzain, Patricia Jeannin, Jean-Paul Grangeon, Sylvie Laumond, Eliane Chungue, Paul M. V. Martin, Antoine Gessain

**Affiliations:** 1 Institut Pasteur, Unité d’Epidémiologie et Physiopathologie des Virus Oncogènes, Département de Virologie, Paris, France; 2 CNRS, UMR 3569, Paris, France; 3 Institut Pasteur de Nouvelle-Calédonie, Nouméa, Nouvelle-Calédonie, France; 4 Centre Hospitalier Territorial de Nouvelle-Calédonie, Nouméa, Nouvelle-Calédonie, France; 5 Direction des Affaires Sanitaires et Sociales, Service de Santé Publique, Nouméa, Nouvelle-Calédonie, France; George Mason University, UNITED STATES

## Abstract

**Background:**

Human T-Lymphotropic Virus type 1 (HTLV-1) is endemic among people of Melanesian descent in Papua New Guinea, Solomon Islands and Vanuatu, and in Indigenous populations from Central Australia. Molecular studies revealed that these Australo-Melanesian strains constitute the highly divergent HTLV-1c subtype. New Caledonia is a French overseas territory located in the Southwest Pacific Ocean. HTLV-1 situation is poorly documented in New Caledonia and the molecular epidemiology of HTLV-1 infection remains unknown.

**Objectives:**

Studying 500 older adults Melanesian natives from New Caledonia, we aim to evaluate the HTLV-1 seroprevalence and to molecularly characterize HTLV-1 proviral strains.

**Study design:**

Plasma from 262 men and 238 females (age range: 60–96 years old, mean age: 70.5) were screened for anti-HTLV-1 antibodies by particle agglutination (PA) and indirect immunofluorescence assay (IFA). Serological confirmation was obtained using Western blot assay. DNAs were extracted from peripheral blood buffy coat of HTLV-1 seropositive individuals, and subjected to four series of PCR (LTR-gag; pro-pol; pol-env and tax-LTR). Primers were designed from highly common conserved regions of the major HTLV-1 subtypes to characterize the entire HTLV-1 proviral genome.

**Results:**

Among 500 samples, 3 were PA and IFA positive. The overall seroprevalence was 0.6%. The DNA sample from 1 New Caledonian woman (NCP201) was found positive by PCR and the complete HTLV-1 proviral genome (9,033-bp) was obtained. The full-length HTLV-1 genomic sequence from a native woman from Vanuatu (EM5), obtained in the frame of our previous studies, was also characterized. Both sequences belonged to the HTLV-1c Australo-Melanesian subtype. The NCP201 strain exhibited 0.3% nucleotide divergence with the EM5 strain from Vanuatu. Furthermore, divergence reached 1.1% to 2.9% with the Solomon and Australian sequences respectively. Phylogenetic analyses on a 522-bp-long fragment of the gp21-*env* gene showed the existence of two major clades. The first is composed of strains from Papua New Guinea; the second includes strains from all neighboring archipelagos (Solomon, Vanuatu, New Caledonia), and Australia. Interestingly, this second clade itself is divided into two sub-clades: strains from Australia on one hand, and strains from Solomon Islands, Vanuatu and New Caledonia on the other hand.

**Conclusions:**

The HTLV-1 seroprevalence (0.6%) in the studied adult population from New Caledonia appears to be low. This seroprevalence is quite similar to the situation observed in Vanuatu and Solomon Islands. However it is very different to the one encountered in Central Australia. Taken together, these results demonstrated that Australo-Melanesia is endemic for HTLV-1 infection with a high diversity of HTLV-1c strains and a clear geographic clustering according to the island of origin of HTLV-1 infected persons.

## Author Summary

The human T-lymphotropic virus type 1 (HTLV-1) infects at least 5 to 10 million individuals worldwide. In Australo-Melanesia, a south Pacific region including Papua New Guinea, Solomon Islands, Vanuatu archipelago and Australia, previous studies have shown that HTLV-1 is present in limited remote areas among few ancient Aboriginal populations. The molecular characterization of the HTLV-1 viruses present in such Indigenous individuals indicates that they belong to a specific HTLV-1 genotype called the Australo-Melanesian subtype c. In the present study, we provide evidence that the HTLV-1 endemicity among elderly individuals from New Caledonia is low and quite similar to Vanuatu and Solomon Islands, yet very different to the situation encountered in Central Australia. Furthermore, the newly described full-length HTLV-1 genomic sequences, from two Melanesian natives from New Caledonia and Vanuatu, both belong to the HTLV-1c genotype but are distinct from those of Aboriginal individuals living in neighboring countries. These results suggest that HTLV-1 viral strains were probably introduced among Melanesian native populations during multiple ancient human migration events to these archipelagos.

## Introduction

### Background and objectives

The Human T-lymphotropic virus type 1 (HTLV-1) is a human oncoretrovirus, which causes two major diseases: adult T-cell leukemia/lymphoma and tropical spastic paraparesis/HTLV-1-associated myelopathy. This virus infects at least 5 to 10 million people worldwide [1]. Clusters of high endemicity have been described in certain geographic areas and ethnic groups, in particular in southwestern Japan, sub-Saharan Africa, South America, the Caribbean basin and localized areas in Iran and Australo-Melanesia [1]. Seven main HTLV-1 molecular subtypes are currently reported: the Cosmopolitan a-subtype, five African subtypes (b, d-g) and a Melanesian/Australian c-subtype found in Australia and neighboring Melanesian islands such as Papua New Guinea (PNG) and the Solomon and Vanuatu archipelagos. HTLV-1 exhibits a high genetic stability. The polymorphism observed among the viral strains is linked to the geographic origin of the infected individuals. This low genetic drift can be used as a molecular tool to monitor viral transmission and the movements of ancient infected populations [2–4].

In the context of ongoing studies among Aboriginal individuals in Australo-Melanesia [4–6], we extended our researches on HTLV-1 to native individuals from New Caledonia archipelago.

Therefore, the detection of HTLV-1 molecular variants from southwestern Pacific territories may constitute a powerful epidemiological and phylogenetic tool to reveal the ancient spread of this oncogenic virus, whose presence in this region has not yet been fully elucidated.

The aim of the present study was hence to characterize the HTLV-1 strains that infect Melanesian natives from New Caledonia, and to compare them with those previously characterized from Melanesian natives originating from neighboring territories.

## Methods

### Geographic data and studied populations

**Fig 1:**
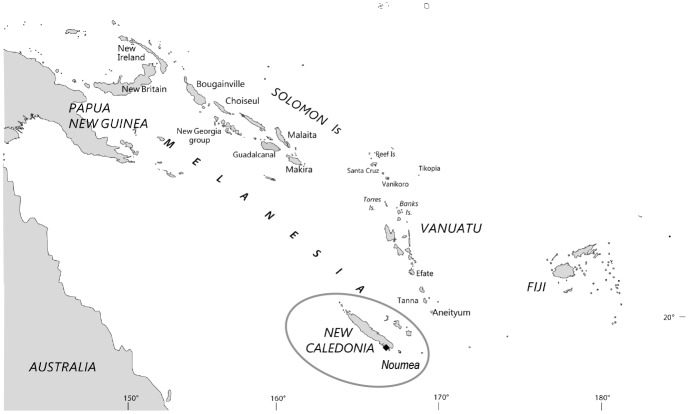
Map of the western Pacific region. The Australo-Melanesia region comprises the islands group extending from Papua New Guinea to New Caledonia including Solomon Islands and Vanuatu archipelago plus Australia, where HTLV-1 epidemiological and clinical situation has been investigated.

New Caledonia (NC), a French overseas territory, is a group of islands located in the Southwest Pacific, east of Australia. (Fig 1). Our work was performed on plasma samples obtained from 500 elderly Melanesian adults, presenting to the blood-taking center at the Institut Pasteur de Nouvelle-Calédonie between July and October 2007. People attending this center usually come from the surroundings of Noumea, the capital city, located in the south of New Caledonia’s main island in the southern province. Briefly, plasma samples were taken from 238 women and 262 men (mean age 70.5 years, range 60–96 years) with the following stratification by age: 48.8% from 60–69 years of age, 39.4% from 70–79 years of age and 11.8% over 80 years of age.

### Ethics statement

This survey received administrative and ethical clearance in New Caledonia from the “Direction des Affaires Sanitaires et Sociales de Nouvelle-Calédonie” and in metropolitan France from the “Comité consultatif sur le traitement de l’information en matière de recherche dans le domaine de la santé” (N° 08.511) and the “Commission nationale de l’informatique et des libertés “(N° 908425). Information regarding the blood collection and the molecular characterization of HTLV-1 proviral strains was provided to the participants presenting to the blood-taking center.

### HTLV-1 serologic analyses

HTLV-1 antibodies in plasma were first detected by a particle agglutination (PA) technique (Serodia HTLV-1, Fujirebio, Tokyo, Japan) at the Institut Pasteur (IP) in New Caledonia and then transferred to the IP in Paris for serological confirmatory assays. An indirect immunofluorescence assay (IFA) using the HTLV-1 and HTLV-2 transformed cell lines MT2 and C19 respectively, was performed. Anti-HTLV-1 antibody titers were determined by successive 2-fold dilutions. All positive samples were further tested by Western blot (WB) assay (HTLV-I/II Blot 2.4, MP Diagnostic, Illkirch, France). A sample with reactivity to the two gag proteins (p19 and p24) and both env-coded glycoproteins (the HTLV-1 recombinant gp46-I peptide [MTA-1] and the HTLV-1/HTLV-2 recombinant [rGD21] protein) was considered to be positive for HTLV-1 antibodies.

### HTLV-1 molecular screening and phylogenetic analyses

High-molecular weight DNA was extracted from peripheral blood buffy coat using the QIAamp DNA Blood Mini Kit (Qiagen Gmbh, Hilden, Germany). DNA samples were subjected to a first polymerase chain reaction (PCR) using human beta-globin specific primers, to ensure that DNA was amplifiable [7]. DNA samples were then subjected to four series of PCR to characterize the entire HTLV-1 proviral genome using primers designed from highly common conserved regions to the major HTLV-1 subtypes. These amplifications have been extensively described [5]. Briefly, we obtained four different HTLV-1 proviral genomic regions: F1, LTR-gag; F2, pro-pol; F3, pol-env and F4, tax-LTR. We also added a proviral DNA obtained from a native woman from Vanuatu (EM5) in the frame of our previous studies, and only partially molecularly characterized [4, 5]. Following electrophoresis, PCR products were purified and sequenced on both strands using a series of specific primers [5]. The Clustal W algorithm (MacVector 6.5 software, Oxford Molecular) was implemented to align forward and reverse sequences of each segment to obtain a consensus sequence of the full HTLV-1 proviral genome. Phylogenetic trees were generated from multiple alignments using HTLV-1 prototypic sequences available in Genbank, and the new sequences generated in this work.

## Results

### HTLV-1 endemicity in the New Caledonian adult population

Seven of the 500 plasma samples studied were tested positive by PA. Among them three were IFA positive with higher immunofluorescence assay titers on MT-2 (HTLV-1) than on C-19 (HTLV-2) cells (Table 1).

**Table 1:** Human T-cell lymphotropic virus type 1 serologic screening tests.

			IFA^¶^ titers		
Virus strain	Age (y)	PA^★^ titers	MT2	C19	WB^§^ pattern
NCP 91	69	1:2,048	1:640	1:80	HTLV-1
NCP 173	65	1:2,048	1:320	1:80	HTLV-1
NCP 201	73	1:4,096	1:1,280	1:160	HTLV-1

Human T-cell lymphotropic virus type 1 (HTLV-1) antibody titers for HTLV-1 seropositive New Caledonian individuals.

^★^PA, particle agglutination;

^¶^ IFA, immunofluorescence assay;

^§^ WB, Western blot.

**Fig 2:**
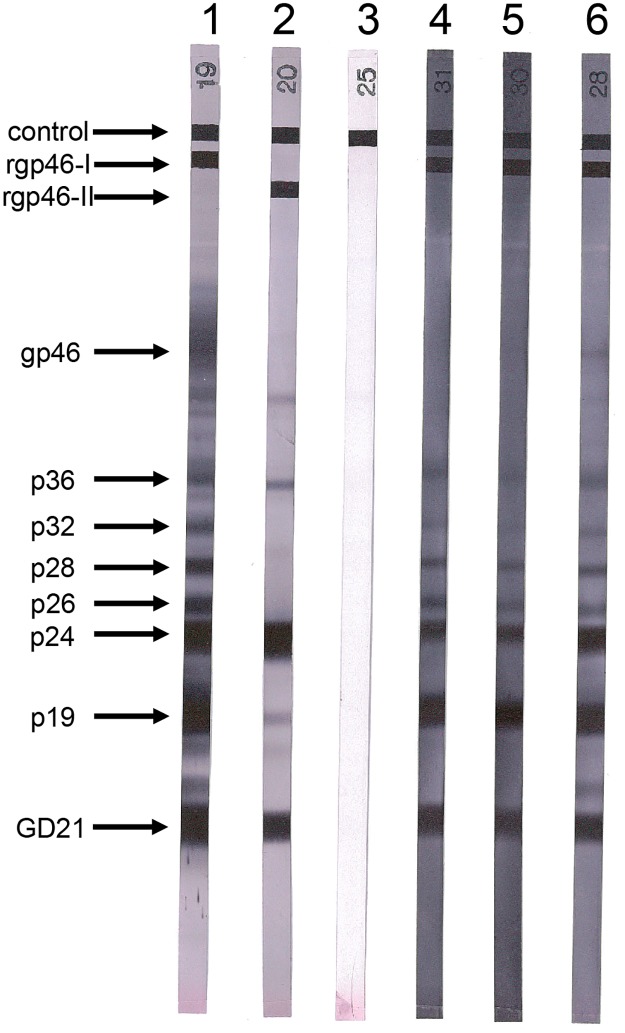
Human T-cell lymphotropic virus type 1 serologic confirmation by Western blot. HTLV-1 seroreactivity patterns obtained by Western blot with recombinant GD21 (common to HTLV-1 and HTLV-2) and two synthetic peptides specific for HTLV-1 (MTA-1) and HTLV-2 (K55). Lane 1, HTLV-1 positive control; lane 2, HTLV-2 positive control; lane 3, HTLV-1/2 negative control; lanes 4–6, plasma samples from the HTLV-1 positive women from New Caledonia (NCP91, NCP173 and NCP201) displaying a strong reactivity to GD21 and to p19, p24, p26, p28, p32, p36 plus rgp46-I (MTA-1).

These three plasma samples exhibited high PA assay titers (≥ 1:2,048) and displayed full reactivity on WB (Fig 2). Taken together, these results revealed a low HTLV-1 prevalence (0.6%, 3/500) in indigenous Melanesian adults. Furthermore, the three positive samples were drawn from women.

### HTLV-1 complete genomic sequence analyses

Amplification of HTLV-1 proviral genome was tested for the 3 HTLV-1-seropositive women (NCP91, NCP173 and NCP201) and a sample from Vanuatu (EM5). Complete HTLV-1 genomic sequences (9,033-bp), derived from 4 PCR fragments of the appropriate size, were obtained for a unique sample from New Caledonia (NCP201; age = 73 years) and the sample from Vanuatu (EM5; age = 76 years). The New Caledonian NCP201 strain exhibited a 0.3% nucleotide divergence with the EM5 strain from Vanuatu. The pairwise comparison of these two new strains with other available complete sequences from Solomon Islands (Mel5) and Australia (Aus-CS, Aus-DF, Aus-NR and Aus-GM) revealed an overall nucleotide polymorphism of up to 2.9% (1.1% to 2.9% respectively) [8].

**Fig 3:**
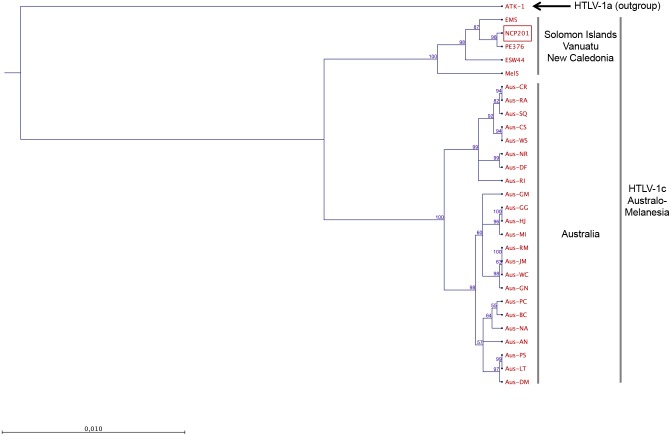
Phylogenetic tree generated with neighbor-joining (NJ) method on a 2,346-bp fragment of the HTLV-1 *gag-tax* concatenated genes for 29 HTLV-1 available sequences including the 2 sequences generated in this work (NCP201 and EM5). HTLV-1 strains were aligned with DAMBE software (version 4.2.13). The final alignment was submitted to the Model test program (version 3.6) to select, according to the Akaike Information Criterion (AIC), the best model to apply to phylogenetic analyses. The selected model was the GTR. The numbers at some nodes of the tree (bootstrap values) were calculated for 1,000 replicates and indicate frequencies of occurrence for 100 trees (bootstrap ≥50%). The branch lengths are drawn to scale with bar indicating 0.01-nucleotide replacement per site. The ATK-1 strain was used as outgroup. The NCP201 and EM5 strains belong to the Australo-Melanesian HTLV-1c subtype and clustered with the strains previously characterized in Vanuatu (ESW44) the Solomon Islands (Mel5). Strains from Central Australia constitute a second clade (Genbank accession nos. KX905202 and KX905203).

To include in our analyses most of the Central Australian sequences characterized to date [5], we performed additional phylogenetic analyses using both neighbour-joining (NJ) and maximum likelihood (ML) methods on the concatenated gag-tax genomic fragment (2,346-bp). First, the results obtained from NJ and ML (data not shown) methods confirmed that the NCP201 strain belongs to the HTLV-1 Australo-Melanesian c-subtype. Second, two clades supported by high bootstrap values (100%) exist within the HTLV-1c subtype. The first clade comprises strains from New Caledonia (NCP201), Vanuatu (EM5, PE376 and ESW44) and Solomon Islands (Mel5), and the second clade includes the sequences from Australia (Fig 3).

### HTLV-1 Gp21-*env* gene fragment analyses

**Fig 4:**
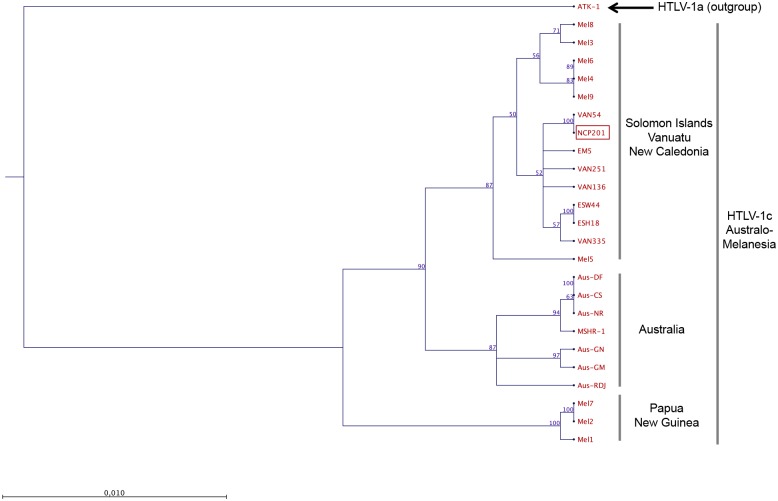
Phylogenetic tree generated with Neighbor-joining (NJ) method on a 522-bp fragment of the HTLV-1 gp-21 *env* gene for 25 HTLV-1 available sequences including the 2 sequences generated in this work (NCP201 and EM5). HTLV-1 strains were aligned with DAMBE software (version 4.2.13). The final alignment was submitted to the Model test program (version 3.6) to select, according to the Akaike Information Criterion (AIC), the best model to apply to phylogenetic analyses. The selected model was the Tamura Nei. Bootstrap values were calculated for 1,000 replicates and indicate frequencies of occurrence for 100 trees (bootstrap ≥50%). The branch lengths are drawn to scale with bar indicating 0.01-nucleotide replacement per site. The ATK-1 strain was used as outgroup. The NCP201 and EM5 strains belong to the “Solomon/Vanuatu/New Caledonian” sub-clade, while the two other “Australian” and “Papua New Guinean” clades exist within HTLV-1c subtype. (Genbank accession nos. KX905202 and KX905203).

As most of available HTLV-1 sequences from PNG were generated on a 522-bp fragment of the gp21-*env* gene, we further analyzed this region to better understand the relationships within the HTLV-1c subtype strains. Thus, the nucleotide homology of the NCP201 strain reached [97.1%-97.3%] with the PNG strains (Mel1, Mel2 and Mel7). Interestingly, the New Caledonian NCP201 strain exhibited a nucleotide similarity range of [99.6%-100%], [99%-99.4%] and [98.1%-98.5%] with the Vanuatu, Solomon Islands and Australian strains respectively (Fig 4). Phylogenetic analyses using the 522-bp fragment revealed the existence of two major clades. The first comprised strains from Papua New Guinea, while the second included strains from all neighboring archipelagos; Solomon Islands, Vanuatu, New Caledonia, and Australia. Interestingly, this latter clade itself is divided into two sub-clades, with strains from Australia on one side, and strains from Solomon Islands, Vanuatu and New Caledonia on the other side (Fig 4). Both clades and sub-clades are supported by high bootstrap values (≥87%).

## Discussion

This study extends to New Caledonia the HTLV-1 endemicity described in native populations from Australo-Melanesia. HTLV-1 seroprevalence (0.6%) in the studied adult population appears to be low but is quite similar in Vanuatu and Solomon Islands. Indeed, previous studies showed that HTLV-1 seroprevalence in Vanuatu archipelago reached 0.74% among individuals aged over 60 years old, while among native Solomon islanders over 50 years old, the reported seroprevalence was 2.3% [4, 9]. This result contrasts with the situation in Australia where HTLV-1 prevalence among Indigenous Australians reached 40% in adults over 50 [10]. Of note, we investigated New Caledonian natives mostly living in the southern part of the main island, and clusters of higher HTLV-1 endemicity may exist elsewhere, as reported in other HTLV-1 endemic areas [11, 12]. Furthermore, some specific risk factors like cultural practices performed in the context of initiation rites, may only be present in populations from Central Australia [10].

From a molecular point of view, the NCP201 sequence belongs to the HTLV-1c subtype, together with the Melanesian strains from Papua New Guinea, Solomon Islands and Vanuatu [4, 6, 13, 14], as well as strains infecting Indigenous individuals from Central Australia [5, 15–17]. Phylogenetic analyses on the gag-tax fragment revealed the existence of two major clades within the HTLV-1c subtype (Fig 3). The first one includes all strains from Australia, and the second one comprises the strains from Vanuatu, New Caledonia and Solomon Islands. Subsequent analyses with the gp21 *env* gene fragment revealed the existence of a PNG clade. Thus, even among HTLV-1c subtype, a clustering according to the geographic origin of the HTLV-1 infected individuals exists.

Though this study is the first demonstration that New Caledonia is endemic for HTLV-1 infection, it has some limitations. The small sample size precluded a detailed epidemiological analysis that could illustrate the geographical dispersal of HTLV-1 infection in the whole New Caledonian archipelago. A further limitation was our inability to molecularly characterize various HTLV-1 strains. This would certainly require HTLV-1-infected individuals exhibiting a higher proviral load. Nevertheless, our findings are consistent with previous studies that have found that HTLV-1 seroprevalence was low among Indigenous Melanesian natives from neighboring archipelagos (e.g. Solomon Islands, Vanuatu).

Current studies combining both host genetics markers and viral analyses on HTLV-1, but also on Kaposi's sarcoma-associated herpesvirus (KSHV)/human herpesvirus 8 (HHV-8), which infects the same aboriginal populations [18], are ongoing to get better insights into the peopling of such Melanesian territories [18–20].
